# Root Illumination Promotes Seedling Growth and Inhibits Gossypol Biosynthesis in Upland Cotton

**DOI:** 10.3390/plants11060728

**Published:** 2022-03-09

**Authors:** Jiayi Zhang, Tianlun Zhao, Kuang Sheng, Yue Sun, Yifei Han, Yiran Chen, Zhiying E, Shuijin Zhu, Jinhong Chen

**Affiliations:** 1 Institute of Crop Science, Zhejiang University, Hangzhou 310058, China; 21916135@zju.edu.cn (J.Z.); tlzhao@zju.edu.cn (T.Z.); 284373@zju.edu.cn (K.S.); 11916039@zju.edu.cn (Y.S.); 11916040@zju.edu.cn (Y.H.); yrchen@zju.edu.cn (Y.C.); 22016027@zju.edu.cn (Z.E.); shjzhu@zju.edu.cn (S.Z.); 2 Hainan Institute, Zhejiang University, Sanya 572025, China

**Keywords:** *Gossypium*, root illumination, seedling growth, gossypol biosynthesis

## Abstract

Gossypol, a terpenoid compound mainly synthesized in the cotton roots, acts as a phytoalexin in protecting the plants from biotic stress. Roots are critical for both the secondary metabolism and the growth of the plant. Light plays an important role in plant growth and material metabolism, however, the effect of root illumination (RI) on the cotton seedling growth and gossypol metabolism remains unclear. In the present study, the cotton genetic standard line TM-1 and four pairs of near-isogenic lines (NILs) were used as materials to study the impact of RI on cotton seedlings. Results showed that, compared with the cotton seedlings cultivated without RI, the photosynthetic rate, leaf area, and dry weight of roots and leaves were significantly increased, while the gossypol content in leaves and roots was significantly reduced in seedlings cultivated with RI. GO and KEGG enrichment analysis of the differentially expressed genes (DEGs) with and without RI both indicated that photosynthesis and terpenoid biosynthesis-related GO terms and pathways were significantly enriched, the expression profile confirmed that RI positively regulated the photosynthesis system and negatively affected the gossypol biosynthesis pathway in roots. This study revealed the effects of RI on seedlings’ growth and gossypol biosynthesis in upland cotton, and provided important insights for the engineering of cotton with low gossypol accumulation.

## 1. Introduction

Cotton is not only an important fiber raw material, but also a source of vegetable oil and vegetable protein. Cottonseed contains protein and oil of a high nutritional value [1], and the protein in cottonseed produced per year could meet the needs of 500 million people [2]. However, due to the presence of gossypol, which is toxic to humans and monogastric animals, the comprehensive utilization of cottonseed is greatly limited [3]. The gossypol also acts as a phytoalexin in cotton, thus playing an important role in protecting the plants from biotic stress [4,5,6,7,8]. Therefore, it is meaningful to research the metabolism of gossypol for the cottonseed utilization and the defense mechanism of cotton plants.

Gossypol is a yellow dimeric sesquiterpene compound, which is synthesized in *Gossypium* and its relative species. There are two optical isomers of gossypol, (+)-gossypol and (−)-gossypol [4], with the second one being more biologically active than the first [9,10,11]. For cotton plants, gossypol is an important phytoalexin not only for resistance to diseases induced by *Rhizoctonia solani*, *Xanthomonas* spp., and *Verticillium dahliae.* [6,7,8] but also for defense against pests including herbivorous insects, in particular, the larvae of *Heliothis virescens* [4,5]. Besides, studies have found that gossypol possesses anticancer potency in the central nervous system’s tumor cell lines, head and neck squamous cell carcinomas, bone marrow cancer, and human breast cancer [9,10,11,12,13], and also could be used as a male contraceptive agent [14,15]. Thus, research on the biosynthesis and decomposition of gossypol is significant for its effective utilization.

Gossypol is mainly synthesized in roots, then transported to the above-ground parts of the plant and stored in the brown punctate pigment glands, which are located on the epidermal tissues of cotton roots, stems, leaves, and seeds [16,17]. The above-ground part also has a certain ability to synthesize gossypol, but it cannot cause significant changes in the gossypol content [17]. The gossypol is synthesized by the mevalonic acid (MVA) pathway, which has been almost elucidated from farnesyl diphosphate (FPP) to gossypol [18]. A series of key enzymes including CDN, CYP706B1, DH1, CYP82D113, CYP71BE79, SPG, CYP736A196, and 2-ODD-1 were characterized [19]. Gossypol is unstable to alkali, heat, and light, and is easily oxidized by them, high temperature conditions and light in vitro can also easily lead to the decomposition of gossypol [20,21]. Therefore, the gossypol samples should be stored at a low temperature and protected from light.

Light is an essential environmental factor that contributes and regulates plant growth and development [22]. Aside from providing the energy for photosynthesis of plants, it also regulates biological processes including seed germination, seedling growth, flowering, and others [23,24]. Direct illumination of roots alters their morphology, cellular, and biochemical responses [25]. In the previous study, it was noticed that light has an effect on the gossypol content during the germination of cotton seeds, and so we wondered if light has an effect on gossypol content and biosynthesis in glanded and glandless cotton seedlings. It was assumed that light has an inhibiting role towards gossypol biosynthesis. Up to now, there are few studies on the effect of light on the biosynthesis of gossypol in cotton. Therefore, an illumination condition for root growth was created in the present study, and the effects of root illumination on the seedling growth and gossypol biosynthesis in different varieties of upland cotton seedlings were investigated.

## 2. Materials and Methods

### 2.1. Materials

The *Gossypium hirsutum* L. accessions, including TM-1 and four pairs of near-isogenic lines (NILs), were used in the present study. The genetic standard line, TM-1, was kindly provided by the USDA-ARS, College Station, Texas, USA. Four pairs of NILs included CCRI12, CCRI12W, CCRI16, CCRI16W, CCRI17, CCRI17W, Coker 312, and Coker 312W, among which CCRI12, CCRI16, CCRI17, and Coker 312 were kindly provided by the Cotton Research Institute, Chinese Academy of Agricultural Sciences. CCRI12W, CCRI16W, and CCRI17W were the glandless NILs of CCRI12, CCRI16, and CCRI17, respectively, and their glandless traits were controlled by the dominant glandless gene ${Gl}_{e}^{2}$, while Coker 312W was the glandless NIL of Coker 312, and its glandless trait was controlled by the recessive glandless genes *gl*_2_ and *gl*_3_. All the above materials were maintained in our laboratory by self-crossing.

### 2.2. Root Illumination

The seeds of the tested material were sown in pots containing the nutrient soil. After the cotyledons were flattened (soil culture for 10 days), the seedlings were transferred to the transparent bucket with complete light transmission and to the black bucket with no light transmission, which were hydroponically cultured in Hoagland’s nutrient solution for 20 days, and the nutrient solution was changed every three days. The pots and buckets were all put in a climatic chamber with the light intensity of 1500 μmol m^−2^ s^−1^, photoperiod of 14 h light period/28 °C, 10 h dark period/24 °C.

### 2.3. Determination of Biomass, Leaf Area and Photosynthesis

After twenty days of culture with and without root illumination, nine seedlings of each treatment group were taken for the measurement of net photosynthetic rate. The third leaf was measured in all seedlings in the climatic chamber with the light intensity of 1500 μmol m^−2^ s^−1^, relative humidity of 60%, and a temperature of 28 °C. The measurement time was settled as 10:00 am, and the Li-6800 Photosynthetic Measurement Instrument (LI-COR, Lincoln, NE, USA) was applied for the measurement. Then, samples were harvested, separated for roots and leaves, taken for measurement of the biomass and leaf area. The leaf area was measured by Leaf Area Survey Instrument YMJ-D (Tuopuyunnong Technology Co., Ltd., Hangzhou, China). Subsequently, the separated roots and leaves were washed by distilled water, dried in the oven at 70 °C for 96 h, and measured for the dry weight.

### 2.4. Extraction and Determination of Gossypol

Fresh samples of leaves and roots from seedlings with and without root illumination were vacuum dried at −40 °C, ground with a high flux tissue grinder, and sieved with a pore diameter of 0.178 mm. Each sample (0.1000 g) was weighed and put into a 10 mL centrifuge tube, then 2 mL derivatization reagent (2% d-aminopropanol: 10% glacial acetic acid: 88% acetonitrile) was added into each sample. The samples were placed in an ultrasonic cleaning machine (30 °C, 240 w) for ultrasonication for 30 min, then heated in a water bath for derivatization (75 °C, 45 min), and centrifuged for 10 min (4 °C, 6000 R min^−1^). Organic microporous filter membrane (0.45 μm) was applied to filter the supernatant into a 10 mL brown volumetric flask, then 2 mL acetonitrile was added for washing the sediment three times and then filtered into the 10 mL brown volumetric flask, and the total supernatant after centrifugation was fixed to 10 mL with acetonitrile. The samples were stored in a refrigerator at −20 °C away from light.

The content of gossypol was determined by high performance liquid chromatography (HPLC). Gossypol standard solution (concentration gradient of 0.00, 0.25, 0.50, 1.00, 2.50, 5.00, 10.00, 25.00, 40.00, 50.00, and 100.00 μg mL^−1^) was set up. The mobile phase was acetonitrile: 10 mM KH_2_PO_4_ (pH = 3) = 80:20 (*v*/*v*). A reverse C18 chromatographic column was used, the column temperature was set at 25 °C, and the UV detection wavelength was set at 238 nm. The samples were measured in triplicates.

### 2.5. Transcriptome Sequencing and Analysis

Roots from TM-1 seedlings with and without root illumination were sampled by liquid nitrogen, and stored at −80 °C. Three biological replicates were set in each treatment. The mRNA was purified from total RNA using poly-T oligo-attached magnetic beads. First strand cDNA was synthesized using random hexamer primer and M-MuLV Reverse Transcriptase, and second strand cDNA synthesis was subsequently performed using DNA Polymerase I and RNase H. Then the cDNA library was constructed by NEBNext Ultra^TM^ RNA Library Prep Kit for Illumina (NEB, Ipswich, MA, USA). Six libraries in total were sequenced by Illumina Hiseq 4000 platform (Novogene Co. Ltd., Beijing, China). Clean reads were obtained by removing reads containing adapter, reads containing ploy-N (N means that the base information cannot be determined) and low-quality reads (bases with Qphred <= 20 accounted for more than 50% of the entire read length) from raw data. All the downstream analyses were based on the clean reads of high quality. The TM-1 reference genome was used for alignment by HISAT2 software [26,27]. The differentially expressed genes were analyzed by DESeq2 software [28], the screening conditions for the FPKM value of DEGs were set as |Fold Change| ≥ 2 and padj ≤ 0.05. The differentially expressed genes were enriched in GO function and KEGG pathway by clusterProfiler software [29].

### 2.6. Statistical Analysis

One-way analysis of variance with the least significant difference (LSD) was applied to determine the significant differences between treatments by using SPSS 22.0. Figures were drawn by GraphPad Prism 8 and TBtools according to the obtained results [30].

## 3. Results

### 3.1. The Effect of RI on Growth of Cotton Seedlings

**Figure 1:**
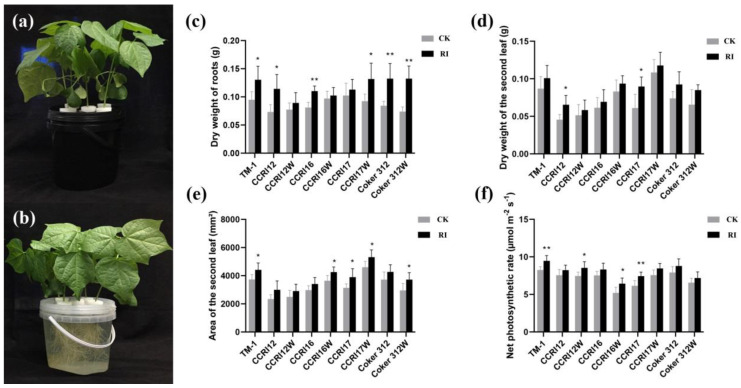
The effect of root illumination on the phenotype of the glanded and glandless cotton seedlings. (**a**) the cotton seedlings without root illumination (CK); (**b**) the cotton seedlings with root illumination (RI); (**c**,**d**) the dry weight of the roots and the second leaf in glanded and glandless cotton with and without RI; (**e**) the area of the second leaf in glanded and glandless cotton with and without RI; (**f**) the net photosynthetic rate in glanded and glandless cotton with and without RI. Error bars in figures represent standard deviation (SD) value (*n* = 9), * indicate significance at *p* < 0.05, ** indicate significance at *p* < 0.01.

The cotton seedlings cultivated with and without root illumination are shown in Figure 1a,b. After 20 days of culture, the dry weight of roots (Figure 1c), dry weight of leaves (Figure 1d), and area of the second leaf (Figure 1e) of RI seedlings were 5.46–79.30%, 8.58–46.81%, and 14.56–27.63% higher than those of control check (CK) in different accessions, respectively. Among them, Coker 312W had the largest increase in dry weight of roots (79.30%, *p* < 0.01), and CCRI17 had the largest increase in dry weight of leaves (46.81%, *p* < 0.05). The second leaf area of CCRI12 increased the most (27.63%, *p* = 0.11) in RI. The four pairs of NILs increased differently in biomass under RI conditions, the average increase of dry weight of roots, dry weight of leaves, and area of the second leaf in the glanded cotton seedlings were 39.84%, 31.98% and 20.31%, respectively, while in the glandless NILs cotton seedlings were 35.66%, 15.96% and 18.76%, respectively. Besides, after the cotton seedlings were cultivated with root illumination, the net photosynthetic rate of the leaves was higher than CK, with an increase of 8.93–23.50% (Figure 1f). Among them, CCRI16W increased the most (23.50%, *p* < 0.05), while the net photosynthetic rate of the leaves in TM-1 and CCRI17 were significantly (*p* < 0.01) higher than those of CK. The average increase of net photosynthetic rate in the glanded cotton seedlings was 12.80%, while in the glandless NILs cotton seedlings the increase was 14.74%. Therefore, it was indicated that RI significantly promoted the growth of cotton seedlings, and the promoting effect on glanded cotton was greater than that on glandless cotton.

### 3.2. The Effect of RI on the Gossypol Content of Cotton Seedlings

**Figure 2:**
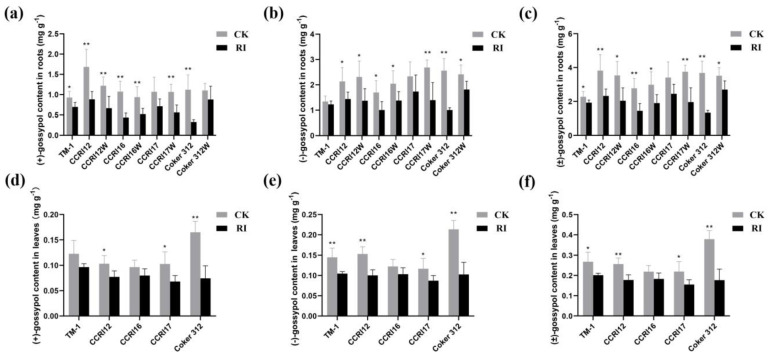
The effect of root illumination on the gossypol content in the glanded and glandless cotton seedlings. (**a**–**c**) the (+)-gossypol, (−)-gossypol, and (±)-gossypol content of the roots in glanded and glandless cotton with and without root illumination (RI). (**d**–**f**) the (+)-gossypol, (−)-gossypol, and (±)-gossypol content of the leaves in glanded and glandless cotton with and without RI. Error bars in figures represent standard deviation (SD) value (*n* = 9), * indicate significance at *p* < 0.05, ** indicate significance at *p* < 0.01.

The (+)-gossypol content in the roots of nine cotton accessions under RI were 20.29–70.79% lower than those in CK (Figure 2a), the decrease of (−)-gossypol content in roots were 8.49–60.75% (Figure 2b), and the decrease of (±)-gossypol content in roots were 15.23–63.81% (Figure 2c). Among them, the (±)-gossypol content in the roots of Coker 312 decreased the most (63.81%, *p* < 0.01). The four pairs of NILs decreased differently in (±)-gossypol content under RI conditions, the average decrease of (±)-gossypol content in the glanded cotton seedlings was 44.78%, while in the glandless NILs cotton seedlings the average decrease was 37.44%. It indicated a greater decrease of gossypol content in the glanded cotton seedlings under RI.

The gossypol content in leaves of glandless cotton accessions was extremely low, thus, the gossypol content was only determined for the five glanded cotton accessions. The results showed that the (+)-gossypol content in leaves of RI was 17.23–54.86% lower than CK (Figure 2d), the decrease of (−)-gossypol content in leaves was 15.51–51.98% (Figure 2e), and the decrease of (±)-gossypol content in leaves was 16.27–53.23% (Figure 2f). Among them, the (±)-gossypol content in leaves of Coker 312 decreased the most with a significant difference (53.23%, *p* < 0.01), while that of CCRI16 decreased the least (16.27%, *p* = 0.09). Thus, it was suggested that RI negatively affected the accumulation of (±)-gossypol content in the leaves and roots of cotton seedlings.

### 3.3. Transcriptome Analysis in Roots of Cotton Seedlings

#### 3.3.1. Sequencing Quality

The quality inspection results of the transcriptome sequencing data showed that the GC content range of samples was 42.44~44.13%, and the ratio of Q30 was more than 93%. After filtering out the unqualified sequences from the original data, the ratio of alignment to the reference genome was 92.11~97.08%, of which more than 88.08% was aligned to a unique position (Table 1), which can be assessed as high-quality data and used for subsequent analysis.

#### 3.3.2. Analysis of Differentially Expressed Genes

**Figure 3:**
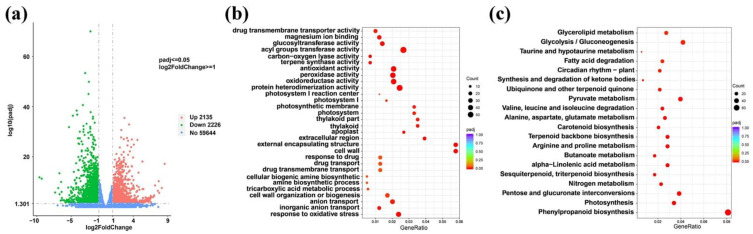
The differentially expressed genes and enrichment analysis of the DEGs under root illumination in TM-1. (**a**) the volcanic map of the DEGs under root illumination (RI), the green dots represent the downregulated genes, the red dots represent the upregulated genes, and the blue represents genes were not significantly differentially expressed, significance is set at padj < 0.05; (**b**,**c**) the GO and KEGG enrichment results of the DEGs under RI.

A total of 4361 differentially expressed genes were identified. Compared with CK, 2135 genes were upregulated and 2226 genes were downregulated in RI (Figure 3a).

#### 3.3.3. GO Enrichment Analysis of Differentially Expressed Genes

GO enrichment analysis was carried out for DEGs, and a total of 72 GO terms were significantly enriched (padj < 0.05), and the top 30 terms with the most significant enrichment are shown in a scatter plot (Figure 3b). It is worth noting that the upregulated genes in RI were significantly enriched in seven photosynthesis related terms, including photosynthesis, photosystem, photosynthetic membrane, photosystem I, photosystem II, thylakoid, and thylakoid membrane. Among the seven terms, more than 90% of the DEGs were significantly upregulated. Besides, all of the downregulated genes were significantly enriched in GO terms of terpene synthase activity, antioxidant activity, peroxidase activity, and oxidoreductase activity. It was also noteworthy that there were 15 DEGs annotated in the GO term of terpene synthase activity, of which 11 genes were downregulated, and this term is related to gossypol biosynthesis.

#### 3.3.4. KEGG Enrichment Analysis of Differentially Expressed Genes

KEGG enrichment analysis was carried out for DEGs, and a total of 29 KEGG pathways were significantly enriched (padj < 0.05), and the most significantly enriched 20 metabolic pathways are shown on a scatter plot (Figure 3c). The upregulated genes in RI were significantly enriched in metabolic pathways such as tryptophan metabolism, carotenoid biosynthesis, porphyrin and chlorophyll metabolism, and photosynthesis pathways. In the photosynthesis pathway, 25 of the 30 DEGs were upregulated. The downregulated genes in RI were significantly enriched in glycolysis/gluconeogenesis, terpenoid backbone biosynthesis, sesquiterpenoid and triterpenoid biosynthesis, and other pathways. In the pathways of terpenoid backbone biosynthesis and sesquiterpenoid and triterpenoid biosynthesis, 20 of the 25 and 12 of the 15 DEGs were downregulated in RI, respectively. Therefore, the KEGG enrichment result was in accordance with the GO analysis result, which indicated that RI had a positive effect on the photosynthesis and a negative impact on the terpenoid biosynthesis.

### 3.4. Photosynthesis Pathway Response to RI

**Figure 4:**
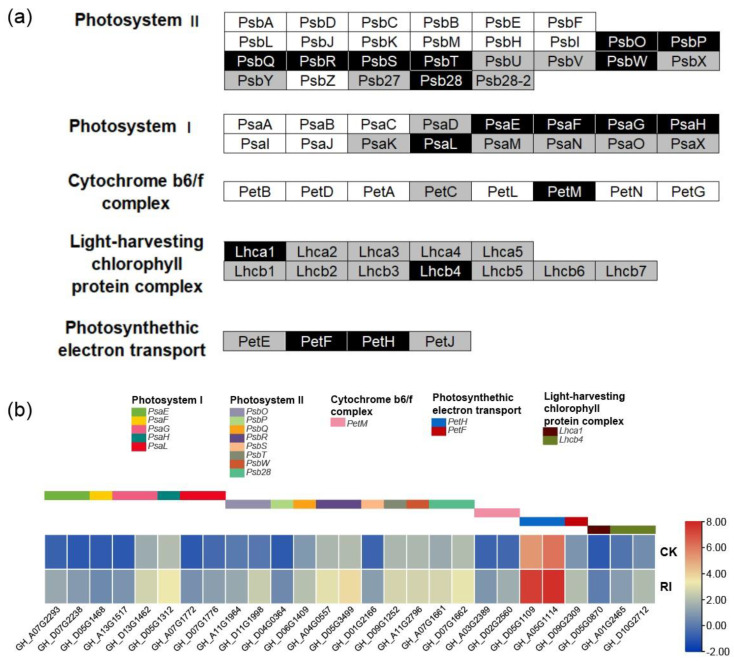
The significantly upregulated photosynthesis genes under root illumination. (**a**) the genes participating in photosynthesis pathway including photosystem I, photosystem II, cytochrome b6/f complex, light-harvesting chlorophyll protein complex (LHC) and photosynthetic electron transport, the dark boxes represent the significantly upregulated nuclear-encoded genes under RI (padj < 0.05), the gray boxes represent the rest of the nuclear encoded genes which are not significantly differentially expressed, and the white boxes represent the plastid-encoded genes in the pathway; (**b**) the expression heatmap of the significantly upregulated genes under RI. The color scale is located at right, the color indicates the average Fragments Per Kilobase per Million (FPKM) of three replications, and significance is set at padj < 0.05.

According to the results of the GO enrichment analysis and KEGG enrichment analysis, it was found that the upregulated genes in roots were significantly related to the photosynthesis system. Therefore, the expression levels of all the photosynthesis genes were investigated (Figure 4a) and the significantly upregulated genes under RI are shown in an expression heatmap (Figure 4b). Among all the genes participating in the photosynthesis pathway, it was figured out that *PsaE*, *PsaF*, *PsaG*, *PsaH*, and *PsaL* were significantly upregulated in photosystem I, *PsbO*, *PsbP*, *PsbQ*, *PsbR*, *PsbS*, *PsbT*, *PsbW*, and *Psb28* were significantly upregulated in photosystem II, *PetM* was significantly upregulated in the cytochrome b6/f complex, *Lhca1* and *Lhcb4* were significantly upregulated in the light-harvesting chlorophyll protein complex, *PetF* and *PetH* were significantly upregulated in the photosynthetic electron transport (Figure 4a). The expression levels of the plastid-encoded genes were not investigated in the study.

### 3.5. Gossypol Biosynthesis Pathway Response to RI

**Figure 5:**
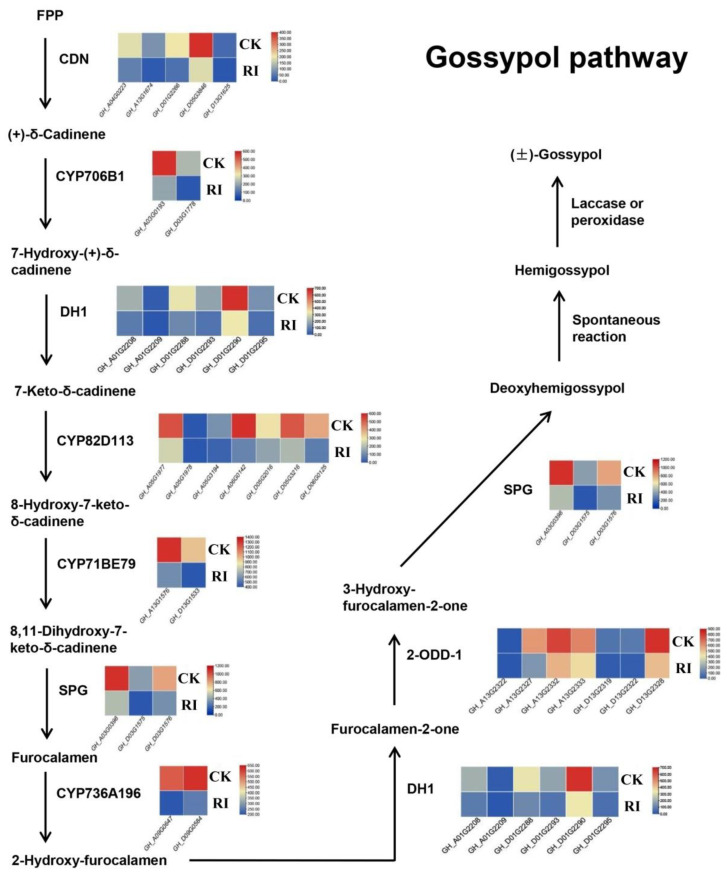
The significantly downregulated genes of the gossypol biosynthesis pathway under root illumination. The expression heatmap indicates the expression of the significantly downregulated genes under root illumination (RI). The color scale is located at the right, the color indicates the average Fragments Per Kilobase per Million (FPKM) of three replication, and significance is set at padj < 0.05.

According to the results of the GO enrichment analysis and KEGG enrichment analysis, it was found that the downregulated genes in RI were significantly related to terpene biosynthesis, which was closely related to gossypol biosynthesis. Therefore, the expression levels of all the gossypol biosynthesis genes were investigated and shown in an expression heatmap (Figure 5). In the gossypol biosynthesis pathway, as expected, all the key genes of the known reactions from FPP to deoxyhemigossypol were significantly downregulated in RI (padj < 0.01), including *CDN*, *CYP706B1*, *DH1*, *CYP82D113*, *CYP71BE79*, *SPG*, *CYP736A196*, and *2-ODD-1*.

## 4. Discussion

Plants could be divided into the above ground parts, including the shoots and leaves and the underground part, i.e., the roots. The above ground parts mainly function to produce carbohydrates and energy by the process of photosynthesis, which is primarily affected by light intensity and quality [25]. The roots mainly function to absorb water and nutrients that are then transported via the vascular tissues towards the rest of the plant for biological processes [31]. Moreover, the roots could also synthesize many critical metabolites for the regulation of plant development, and some specific compounds including nicotine and gossypol are synthesized in the roots of tobacco and cotton, respectively [16,17,32]. However, the biosynthesis process of these metabolites could be affected by many environmental factors. The accumulation of nicotine could be induced quickly by high temperatures in tobacco roots [33]. In the present study, for the first time we showed that the gossypol biosynthesis in cotton roots could be inhibited by RI, and the accumulation of gossypol content in leaves and roots was significantly reduced.

The primary contribution of light is for the photosynthesis system of plants, which includes Photosystem I, Photosystem II, Cytochrome b6/f complex, Photosynthetic electron transport, and F-type ATPase. It is well known that the main photosynthesis parts are the above ground parts, while the root system possesses low photosynthesis efficiency due to the little light that exists in the soil [34]. In this study, RI was performed to research on its effect, and it was discovered that RI improved the net photosynthetic rate in leaves of all accessions, and the difference between RI and CK in four accessions reached a significant level. Besides, the transcriptome analysis in roots revealed that the *PsaE*, *PsaF*, *PsaG*, *PsaH*, and *PsaL* in Photosystem I were significantly upregulated; the *PsbO*, *PsbP*, *PsbQ*, *PsbR*, *PsbS*, *PsbT*, *PsbW*, and *Psb28* in Photosystem II were significantly upregulated; the *PetM* in Cytochrome b6/f complex was significantly upregulated; *Lhca1* and *Lhcb4* in the light-harvesting chlorophyll protein complex were significantly upregulated; *PetF* and *PetH* in the photosynthetic electron transport were significantly upregulated. Therefore, we speculated that RI could positively regulate the photosynthesis efficiency in roots, however the transcriptional analysis in leaves needs to be performed in a further study to see if the DEGs are enriched in photosynthesis. However, the mechanism of how RI regulated the photosynthesis of leaves remains unclear. This might be mediated by phytohormones such as auxin, because light and auxin signaling cross-talk could program the photosynthesis efficiency to promote plant growth [35,36]. Subsequently, it was also found that the biomass, including the dry weight of roots and leaves, was significantly increased and the leaf area also significantly enlarged. Thus, this study provided experimental evidence that roots exposed to light could promote the seedling growth by regulating the photosynthesis pathway in cotton.

Gossypol is a specific compound which mainly synthesized in the root system of cotton and its relative species, and was then transported to each part of the pigment glands in plants [16,17,37]. According to the existence of pigment glands, which are the storage tissue of gossypol, cotton could be classified into glanded, glandless, and delayed gland morphogenesis cotton. Due to the main element of the pigment glands, gossypol, glanded cotton is more resistant to pests and diseases than the others [4,5,6,7,8]. Moreover, whilst gossypol limited the utilization of cottonseeds for food purposes, which contain superior oil and protein, it did have application in medicine for anti-cancer and contraceptive uses [1,9,10,11,12,13,14,15]. Therefore, it is of great significance to investigate the gossypol biosynthesis in cotton. Here in this study, it was found that RI significantly decreased the contents of (+)-gossypol and (−)-gossypol in roots, whether of glanded or glandless cotton. Besides, the accumulation of (+)-gossypol and (−)-gossypol in leaves of glanded cotton also decreased, while no gossypol content was detected in the leaves of glandless cotton. Furthermore, transcriptome analysis of the roots also showed that the genes encoding key enzymes in the gossypol pathway, including *CDN*, *CYP706B1*, *DH1*, *CYP82D113*, *CYP71BE79*, *SPG*, *CYP736A196*, and *2-ODD-1* were all significantly downregulated. Therefore, it was indicated that RI could inhibit the gossypol biosynthesis in the roots of glanded and glandless cotton. In addition, the biosynthesis of (+)-gossypol and (−)-gossypol were all inhibited. However, the mechanism of light regulation of the gossypol biosynthesis in cotton needs further study. It may be that the molecular mechanism of RI-mediated gossypol biosynthesis inhibition depends on the sensitivity to light of one or more biosynthetic enzymes. Generally, the present study revealed that light could inhibit the gossypol biosynthesis in cotton and provides a theoretical basis for understand the light impact on the metabolism of compound synthesizes in the root system.

## 5. Conclusions

In summary, root illumination could significantly promote the growth of seedlings and inhibit gossypol biosynthesis, both in glanded and glandless cotton. (1) Root exposure to light could upregulate the genes’ expression of the photosynthetic pathway in roots. The photosynthesis efficiency in leaves was positively regulated by root illumination, which promoted the growth of cotton seedlings. Roots may function as a supplement to the above ground parts in promoting seedling growth. Then, subsequently, the biomass of the roots and leaves were increased, and the leaf area was enlarged. (2) Root exposure to light significantly downregulated the expression level of genes participating in the gossypol biosynthesis pathway, and the accumulation of (+)-gossypol and (−)-gossypol in roots and leaves were all decreased. Further research should focus on the mechanism of the root illumination effects on the seedling growth and gossypol biosynthesis by integrating methods such as transcription, signal transduction, and function verification. Overall, this study provided evidence for understanding the effect of root illumination on seedling growth and gossypol biosynthesis in cotton, and provided a theoretical basis for revealing the light impact on root system.

## Figures and Tables

**Table 1:** Statistics and quality testing of transcriptome sequencing data.

Sample	Raw Reads (bp)	Clean Reads (bp)	Q30 (%)	GC Content (%)	Mapped Rate (%)	Uniquely Mapped Rate (%)
CK_1	49,818,318	47,615,882	93.66	42.44	97.08	92.09
CK_2	46,293,350	43,363,002	93.87	42.52	97.05	91.81
CK_3	49,280,916	48,100,220	93.1	43.2	96.88	92.38
RI_1	48,019,602	46,918,760	93.56	43.85	94.14	89.96
RI_2	45,340,188	44,252,532	93.72	44.13	92.11	88.08
RI_3	47,859,738	46,815,136	93.91	43.76	93.91	89.68

## Data Availability

All transcriptomic sequencing data have been deposited in the NCBI Sequence Read Archive under accession number PRJNA800639. Other data are contained within the article.
